# Neuromuscular blockade of atracurium in permissive hypercapnic versus normocapnic swine undergoing laparoscopy

**DOI:** 10.1371/journal.pone.0200439

**Published:** 2018-07-06

**Authors:** Luca Bellini, Giulia Maria De Benedictis

**Affiliations:** Department of Animal Medicine, Production and Health, University of Padua, Legnaro, Italy; University of Bari, ITALY

## Abstract

Neuromuscular blocking agents (NMBAs) are commonly used in experimental laparoscopy in swine undergoing carbon dioxide pneumoperitoneum. Hypercapnia may be present and may prolong NMBAs’ pharmacologic activity. The aim of this study is to evaluate the effect of permissive hypercapnia on the neuromuscular blockade of atracurium in swine. Six Large White swine weighing 30.5 ± 1.6 kg were sedated with intramuscular ketamine and medetomidine, after which anaesthesia was induced with propofol and maintained with sevoflurane. Atracurium 0.4 mg/kg was administered intravenously and the neuromuscular block monitored by acceleromyography during normocapnic and hypercapnic conditions (PaCO_2_ range 35–45 mmHg and 60–70 mmHg, respectively). Onset time and time to reach a train of four ratio (TOFR) of 0.7 and 0.9 were recorded. Cardiorespiratory parameters, electrolytes and acid-base status were measured under both conditions. Onset time was similar between the two conditions. Time to reach a TOFR of 0.7 and 0.9 (duration of the neuromuscular block) was longer in hypercapnic compared to normocapnic animals being 1325 ± 300 vs 855 ±111 (*p* = 0.002) and 1823 ± 434 vs 1218 ± 210 seconds (*p* = 0.005), respectively. Three hypercapnic swine had a TOF count of 2 and 1 instead of a count of 4 with fade. Permissive hypercapnia was associated with a decrease in pH from 7.444 ± 0.039 to 7.257 ± 0.025 (*p* < 0.001). No differences were observed for heart rate, end-tidal concentration of sevoflurane, body temperature and arterial haemoglobin saturation. Nonetheless, hypercapnic swine had a statistically significant increase in mean arterial pressure (*p* = 0.020) and plasma potassium concentration (*p* = 0.003). The values of PaCO_2_ achieved during hypercapnia were well tolerated in swine undergoing CO_2_ pneumoperitoneum for laparoscopy. Permissive hypercapnia increased the duration of the atracurium effect and caused an increase in the intensity of the neuromuscular block in few swine.

## Introduction

The use of non-depolarizing neuromuscular blocking agents (NMBAs) is common during experimental laparoscopic procedures in swine [1–5]. Acceleromyography to measure the train of four ratio (TOFR) has recently been described to monitor neuromuscular blockade (NMB) in pigs [5]. During pneumoperitoneum the insufflation of carbon dioxide (CO_2_) into the abdominal cavity leads to gas absorption and hence hypercapnic acidemia that may not be controlled with mechanical ventilation [1,4,6]. Moreover, aggressive ventilator setting to manage hypercapnia may damage the lung. Besides, moderate increases of CO_2_ arterial partial pressure (PaCO_2_) associated with a pH between 7.2 and 7.3 are often tolerated unless obvious contraindications are present [7]. Moderate or permissive hypercapnia has also been associated with a protective effect towards ventilator-induced inflammation and simultaneously improves hemodynamic function [8].

Atracurium is a NMBA of the benzylisoquinoline class with an intermediate duration of action. The rate-limiting step in the liver-independent degradation pathway of atracurium is the Hofmann elimination, a process dependent on plasma pH and temperature [9]. This makes atracurium advantageous during surgeries in which kidney or liver function may be compromised. In swine, the dose reported for atracurium ranges between 0.6 and 2 mg/kg and this produces a moderate to deep NMB with increasing dose [9–11]. No reports evaluate the shallow NMB obtained with atracurium doses of less than 0.6 mg/kg in swine. Moreover, a recent study suggests that a good surgical operating condition may be achieved with low doses of NMBAs, especially if volatile anaesthetics are used [12].

Duration and intensity of the atracurium NMB are influenced by arterial pH in cats with experimentally induced hypercapnia under chloralose anaesthesia [13]. No studies describe the effect of hypercapnic acidosis due to CO_2_ pneumoperitoneum on the pharmacological effect of atracurium in swine. The aim of this study was to quantify the duration and the intensity of the NMB after 0.4 mg/kg of atracurium during normocapnia versus permissive hypercapnia in swine undergoing laparoscopic unilateral nephrectomy. The dose of atracurium was chosen on the base of a dose-response study that reported a 40% decrease in the response to single twitch usually associated to a TOFR of 4 with fade after a single dose of 0.4 mg/kg [11].

## Materials and methods

### Animals

Six female Large White swine weighing 30.5 ± 1.6 kg were studied in a cross-over study. The study was performed with the approval of the Animal-welfare Body of the University of Padua (OPBA authorization number 8/2016) and the Italian Ministry of Health (authorization number 828/2016), according to the European Directive (2010/63/EU) and Italian regulations (Legislative Decree 26/2014). Animals underwent a left unilateral nephrectomy under laparoscopy as part of another terminal study. The pigs were provided by a conventional breeding farm and housed in an authorized laboratory animal facility at the University of Padua.

Laparoscopy was performed with the animals in right lateral recumbency and the abdomen was inflated with CO_2_ up to an intraabdominal pressure of 12–14 mmHg.

### Anaesthesia

Swine were sedated intramuscularly in the epaxial muscle of the neck with medetomidine (Domitor; Orion Corporation; Finland) 15 μg/kg and ketamine (Ketavet 100; Intervet; Italy) 7 mg/kg, drawn in the same syringe immediately before injection. Ten minutes later swine were moved into the pre-surgical preparation area. A 22-gauge, 25 mm long, over the needle catheter (Delta Ven; DeltaMed Spa; Italy) was inserted aseptically into the metacarpal vein and propofol (Propofol Kabi; Fresenius Kabi Italia s.r.l.; Italy) was administered until the laryngeal reflex disappeared. After endotracheal tube insertion, pigs were moved to the surgery theatre and anaesthesia was maintained with sevoflurane (Sevorane; Abbott; Italy) via a circle breathing system. Morphine (Morfina cloridrato Molteni; L Molteni & C. dei F.lli Alitti; Italy) was injected as intraoperative analgesic at 0.3 mg/kg into the quadriceps muscle. Intramuscular administration was repeated if the procedure lasted more than 4 hours. Respiratory rate was adjusted with a volume-controlled ventilator (Datex-Ohmeda 7900 SmartVent; GE Healthcare; Finland), set to deliver a tidal volume of 10 ml/kg. A mixture of oxygen and air 1:1 was delivered with a total fresh gas flow set initially to 4 L/min and then to 1 L/min. The femoral artery was cannulated with a 20-gauge, 32 mm long, over the needle catheter (Delta Ven; DeltaMed Spa; Italy) for direct blood pressure measurement and arterial blood sample collection. Heart rate (HR), invasive systemic arterial blood pressure, side stream end-tidal carbon dioxide (Pe’CO_2_), end-tidal sevoflurane concentration (Fe’SEVO) and oesophageal temperature were displayed continuously on a multiparameter monitor (Datex-Ohmeda S/3 Compact Anesthesia Monitor; GE Healthcare; Finland). Sodium chloride 0.9% solution (Sodio cloruro S.A.L.F. 0.9%; Samed; Italy) was infused throughout the procedure at 5 mL/kg/hour.

For arterial blood gas analysis, 1 mL of blood was collected from the femoral arterial catheter into a heparinized 1 mL syringe (Marquest; Vital signs Inc; Englewood; CO; USA). Before sample collection, 3 mL of blood were removed to avoid dilution of the sample. The analyser (ABL series 700 XP; Radiometer Medical ApS; Denmark) used approximately 200 μL of blood and the samples was analysed immediately after collection.

#### Neuromuscular monitoring

Neuromuscular function was assessed using an acceleromyograph (TOF-Watch®; Organon Ltd; Ireland). The pigs’ skin over the medial surface of the left forelimb was shaved, cleaned, degreased and dried. Two surface stimulation electrodes were placed over the left ulnar nerve with a distance between the centres of 5 cm and the negative electrode was connected distally. The acceleration transducer was taped distally on the palmar side of the hoof in the cleavage between two toes. The train-of-four (TOF) stimulation pattern was elicited with four stimuli delivered at 2 Hz every 15 seconds. The intensity of the NMB was defined as: deep block if the TOF count was zero; moderate block if TOF count was 1–3; shallow block (TOF count of 4 with fade) if TOFR was ≥ 0.1 [12]. After 10 minutes of repeated TOF stimulation, the TOF-Watch was calibrated according to the manufacturer’s guidelines, and atracurium (Atracurium-Hameln; Hameln Pharmaceuticals gmbh; Germany) 0.4 mg/kg was administered intravenously over 5 seconds.

Neuromuscular block induced by atracurium was studied in each swine under two experimental conditions: in group NormoCO_2_, swine were ventilated to maintain an arterial partial pressure of carbon dioxide (PaCO_2_) between 35–45 mmHg, while in group HyperCO_2_, PaCO_2_ was maintained between 60 and 70 mmHg. If spontaneous ventilation was present, to reach the target PaCO_2_, a bolus of propofol of 3 mg/kg was administered and repeated every 5 minutes until apnoea was achieved. Blood gas analysis was used to confirm the target PaCO_2_ and was repeated every 10 minutes to adjust the ventilator setting to maintain the experimental condition. The NMB was evaluated first in the normocapnic condition until it resolved. The NMB was considered resolved when TOFR returned to 1.00 ± 0.01. After 20 more minutes, the induction of hypercapnia was started to achieve the target PaCO_2_. Subsequently the hypercapnic status was reached and maintained for 5 minutes and the second dose of atracurium was administered.

#### Variables

Under both experimental conditions the following data were recorded: the onset time defined as the time from atracurium injection to the lowest TOFR recorded in each animal (Onset); the times to reach a TOFR of 0.7 (TOFR0.7) and 0.9 (TOFR0.9). During anaesthesia HR, invasive mean arterial blood pressure, Fe’SEVO, Pe’CO_2_ and oesophageal body temperature were recorded. Arterial blood sample was collected and pH, PaO_2_, PaCO_2_, arterial oxygen saturation (SaO_2_), bicarbonate, base excess, lactate, sodium, chloride, potassium and calcium were recorded.

### Statistical analysis

Continuous variables were tested for normality with a Kolmogorov-Smirnov test and, if normally distributed, were expressed as mean ± standard deviation otherwise they were reported as median (min-max). Physiological variables, measured before atracurium injection and when the TOFR was 0.9, were averaged for each experimental condition and used for the analysis (S1 File). A paired Student’s t-test or a Mann Whitney test when appropriate, were used to detect differences among the mean of groups. To identify a mean difference in block duration between experimental conditions of 5 minutes with a standard deviation of 2 minutes, a minimum of 6 animals should be enrolled to obtain a power of 0.90 and an α of 0.05. Analyses were performed with a statistical package (GraphPad Prism 6.0, La Jolla, CA, USA) and *P* < 0.05 was considered statistically significant.

#### Results

The target PaCO_2_ was reached in all the swine for both conditions and in all animals the experimental procedure could be completed. Clamping of the left renal artery and vein was completed in 124 ± 14 minutes from the first dose of atracurium. Spontaneous ventilation was never observed and hence propofol boluses were not administered. Table 1 shows the mean of intraoperative variables for group NormoCO_2_ and HyperCO_2_. No differences were observed between treatment groups for HR (*P* = 0.515), Fe’SEVO (*P* = 0.883) and oesophageal temperature (*P* = 0.227). Group HyperCO_2_ showed a statistically significant higher mean arterial blood pressure compared to treatment group NormoCO_2_: 84 ± 9 and 69 ± 11 mmHg (*p* = 0.031), respectively.

**Table 1:** Cardiovascular variables, fraction of expired sevoflurane and oesophageal temperature of swine undergoing carbon dioxide pneumoperitoneum, maintained normocapnic (PaCO_2_ 35–45 mmHg) or hypercapnic (PaCO_2_ 60–70 mmHg), which received a single bolus dose of 0.4 mg kg^-1^ of atracurium.

Variables	NormoCO_2_	HyperCO_2_	*p* value
HR (beats/min)	98 ± 4	102 ± 12	0.515
MABP (mmHg)	69 ± 11	84 ± 9	**0.031**
Fe’SEVO (vol%)	2.3 ± 0.3	2.3 ± 0.1	0.883
Temperature (°C)	37.3 ± 0.4	37.7 ± 0.8	0.227

NormoCO_2_, swine maintained normocapnic; HyperCO_2_, swine maintained hypercapnic; HR, heart rate; MABP, mean arterial blood pressure; FE’SEVO, end-tidal fraction of sevoflurane.

Table 2 lists the means of the blood gas analysis and PE’CO_2_ data collected during each phase of the experiment. During the hypercapnic phase the average arterial pH was lower and both the PaCO_2_ and PE’CO_2_ were higher than during the normocapnic phase. PaO_2_ was not statistically different between groups although a decrease was observed in swine under hypercapnic condition (*P* = 0.063). No other difference was detected among groups for blood gas parameters, but plasma concentration of potassium was significantly higher during the hypercapnic period than during normocapnia.

**Table 2:** Mean arterial blood gas, end-tidal CO_2_, acid-base, and electrolyte variables determined in swine undergoing carbon dioxide pneumoperitoneum and being maintained either normocapnic (PaCO_2_ 35–45 mmHg) or hypercapnic (PaCO_2_ 60–70 mmHg) after having been administered a single IV bolus dose of 0.4 mg/kg of atracurium.

Variables	NormoCO_2_	HyperCO_2_	*p* value
pH	7.444 ± 0.039	7.257 ± 0.025	**<0.001**
PaCO_2_ (mmHg)	39.1 ± 3.7	64.6 ± 4.8	**<0.001**
PE’CO_2_ (mmHg)	41 ± 5	68 ± 2	**<0.001**
PaO_2_ (mmHg)	205 ± 46	121 ± 18	0.063
SaO_2_ (%)	99.5 ± 0.6	98.2 ± 1.3	0.054
Bicarbonate (mmol/l)	25.6 ± 2.3	27.2 ± 1.7	0.270
Base excess (mmol/l)	1.7 ± 2.6	0.2 ± 1.8	0.294
Lactate (mmol/l)	0.9 ± 0.4	0.6 ± 0.1	0.125
Na (mmol/l)	142 ± 3	141 ± 3	0.601
K (mmol/l)	3.6 ± 0.2	4.0 ± 0.1	**0.003**
Ca (mmol/l)	1.0 ± 0.1	1.0 ± 0.1	0.657
Cl (mmol/l)	106 ± 5	104 ± 5	0.658

PaCO_2_, arterial partial pressure of carbon dioxide; NormoCO_2_, swine maintained normocapnic; HyperCO_2_, swine maintained hypercapnic; PE’CO_2_, end-tidal partial pressure of carbon dioxide; PaO_2_, arterial partial pressure of oxygen; SaO_2_, arterial oxygen saturation; Na, sodium; K, potassium; Ca, calcium; Cl, chloride.

**Fig 1:**
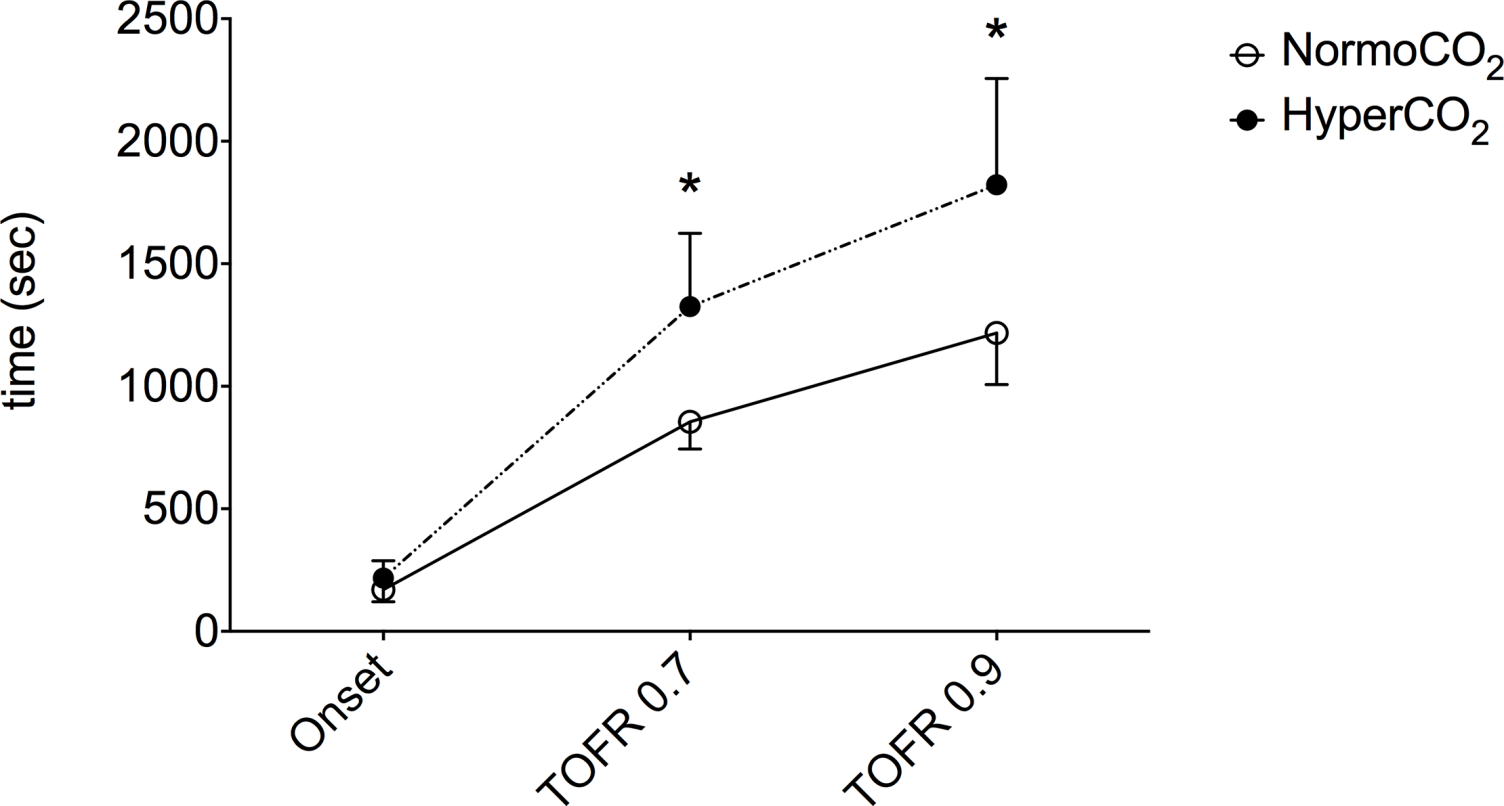
Comparison of time to reach the lowest TOFR from atracurium injection (Onset), the times to reach a TOFR of 0.7 (TOFR 0.7) and 0.9 (TOFR 0.9) between swine maintained normocapnic (PaCO_2_ 35–45 mmHg) or hypercapnic (PaCO_2_ 60–70 mmHg). * P value < 0.05. NormoCO_2_ swine maintained normocapnic, HyperCO_2_ swine maintained hypercapnic.

Onset time was not different between groups (*P* = 0.196) although in hypercapnic swine the time to reach the TOFR of 0.7 and 0.9 was significantly longer than during normocapnia (Fig 1). In the normocapnic group, the TOFR ranged between 0.12 and 0.32 and no animal reached a moderate block. In the hypercapnic group the NMB was deeper; a moderate block with TOF count of 2 and 1 was observed in two and one animals respectively while 3 swine showed a TOFR between 0.15 and 0.26.

## Discussion

The results of this study show that hypercapnic acidosis induced by pneumoperitoneum during laparoscopic surgery prolonged the duration of action of atracurium in swine. The hypercapnic status was also associated with a deeper NMB in three animals that showed a TOF count of 1 or 2 during hypercapnia instead of a TOF of 4 with fade as in normocapnic condition. The duration of action of atracurium is dose dependent in dogs, cats and Rhesus monkeys [13,14]. Gyermek et al. reported that, in pigs, 2.7 mg/kg of atracurium determined a duration of NMB of 45 minutes [15]. In our study the time needed to reach a TOFR of 0.9 was about 20 minutes from the injection in normocapnic animals and it increased to 30 minutes during hypercapnia.

No study evaluated the NMB caused by atracurium in swine under different acid base status in clinical conditions. Huges and Chapple studied the block of atracurium during respiratory acidosis experimentally induced by 20% CO_2_ inhalation in cats anesthetized with chloralose [13]. Values of pH reached in that study ranged from 6.68 to 7.00; in clinical settings those values are seldom observed during permissive hypercapnia and they require aggressive treatment to restore the physiologic status and avoid complications. Permissive hypercapnia instead is a ventilatory strategy that allows a decrease in arterial pH to about 7.20. This approach is also associated with a reduction of ventilator-induced lung injuries, pulmonary inflammation and injuries of liver and myocardium during ischemia [7,8]. In our study plasma pH during hypercapnia was 7.257 and it caused an increase in the duration of the block as observed in cats [13]. Moreover in 3 hypercapnic swine the intensity of the block was increased, as reported at low pH values, *in vitro*, for different non-depolarizing neuromuscular drugs [16]. On the contrary, in cats, regardless of the acidosis, the block observed was almost complete after a single twitch or a tetanic stimulation, similarly to the block recorded in the normocapnic subject [13]. The discrepancy in the results observed in cats compared to those observed in the current study may be due to the dose of atracurium used. In cats, the atracurium dose was indeed higher than the ED_90_ and it was able to suppress the twitch response. Moreover, cats show a high sensitivity to neuromuscular blockade and, as observed also for other NMBAs, to achieve a deep block, smaller doses are needed than in swine [15].

Increased potassium plasma concentration was associated, *in vitro*, with decreased sensitivity of the NMB caused by d-tubocurarine and pancuronium [17]. Although in our study an increase in potassium during the hypercapnic period was observed, it did not decrease the depth of the block, which seemed instead deeper in some swine. The block appeared to be influenced by pH rather than potassium plasma concentration that, nevertheless, remained among the physiologic range for young swine under anaesthesia [18].

In our study, the onset time was not different between experimental conditions. This variable is usually influenced by the initial dose of the NMBA, its volume of distribution and the hemodynamic status rather than plasma pH [19]. The dose of atracurium used in this study was the same for both experimental conditions, and presumably the volume of distribution unlikely changed over the study. Therefore, the onset time of atracurium depended mainly on the hemodynamic status and muscle perfusion. Although in the hypercapnic condition an increase in arterial blood pressure was present, this may not be clinically relevant and did not cause an increase in the muscle perfusion.

An increase in arterial blood pressure and systemic vascular resistance was reported during CO_2_ pneumoperitoneum in anaesthetised pigs as a result of catecholamine and vasopressin release [1–3]. Moreover, hypercapnia is associated with a sympathetic stimulation that causes an increase in systemic vascular resistance [20]. Although atracurium may be associated with a decrease in systemic blood pressure due to histamine release, 0.6 mg/kg produced no effect on cardiovascular parameters in swine anaesthetized with 1.2 MAC of isoflurane or desflurane [10]. The differences in mean arterial pressure observed in the current study were considered due to CO_2_ pneumoperitoneum rather than the administration of atracurium.

The blood gas analysis during the hypercapnic period showed a decrease in arterial partial pressure of oxygen although no statistic differences were observed between groups. The diaphragmatic compression caused by the pneumoperitoneum may have contributed in impairing ventilation/perfusion matching caused by atelectasis [4]. Despite this ventilatory impairment, the arterial haemoglobin oxygen saturation was maintained within normal limit indicating that the intraabdominal pressure of 12–14 mmHg during permissive hypercapnia was well tolerated in swine.

Sodium, chloride and calcium did not change between groups and they unlikely may have influenced the NMB of atracurium. The blood gas analyser used in this study did not measure magnesium and we cannot exclude that an increase in the plasma magnesium could have be occurred. This electrolyte influences the depth and the duration of the block of cis-atracurium in humans although it shortened the onset time [21]. In our study the onset was not different between groups, therefore, even if the increase in extracellular magnesium could not be ruled out, it should have been minimal. Plasma potassium concentration was higher during the hypercapnic period as observed by other authors during CO_2_ pneumoperitoneum in mechanically ventilated normocapnic swine as a result of acidosis caused by peritoneal absorption of CO_2_ [1].

In this study, plasma concentration of atracurium was not measured and it cannot rule out the possibility that the first dose of atracurium administered was not completely cleared during the hypercapnic phase. The residual atracurium may have had an effect on the outcome of the second experimental condition. Nevertheless Hughes and Chappel (1981) demonstrated that increasing the dose of atracurium, the onset time of the NMB shortens. In our study the onset of the NMB was similar between the two experimental conditions supporting the hypothesis that the effect of the first dose did not influence the block observed during the hypercapnic condition. A second limitation might be represented by the chance that surgery may influence the volume of distribution of atracurium and its pharmacokinetics but this would have also affect the onset time of the NMB [13]. Another limitation is represented by the fact that atracurium was studied initially under normocapnia in all swine. When this study was planned, it was estimated that nephrectomy would have last no longer than 2 hours. Acidosis prolongs the duration of the NMB of atracurium in several species [13] and it was hypothesized that this was true also in pigs, although it was not possible to estimate *a priori* the duration of the NMB during hypercapnic acidosis. For this reason, it was chosen to study initially the condition that would have produced the shortest block in an attempt to reduce the variability due to the surgical procedure. Moreover the clamping of the renal artery and vein was mostly completed 10 minutes before the end of the hypercapnic period so the experimental condition did not substantially change during the second phase of the study.

## Conclusion

The duration of the neuromuscular block produced by a single injection of 0.4 mg/kg of atracurium is increased in swine undergoing laparoscopy under permissive hypercapnia. The depth of the neuromuscular blockade increased in 3 animals compared with the normocapnic condition. Permissive hypercapnia with PaCO_2_ between 60 and 70 mmHg is well tolerated in swine undergoing CO_2_ pneumoperitoneum for laparoscopic nephrectomy.

## Supporting information

S1 File Raw data for onset time of the neuromuscular block, the times to reach a TOFR of 0.7 and 0.9, cardiovascular variables, oesophageal temperature, electrolytes, blood gas analysis and respiratory variables. The excel file includes all raw data measured in swine maintained either normocapnic (PaCO_2_ 35–45 mmHg) or hypercapnic (PaCO_2_ 60–70 mmHg) and that received for each condition atracurium 0.4 mg/kg.
(XLSX)
